# Beyond energy provider: multifunction of lipid droplets in embryonic development

**DOI:** 10.1186/s40659-023-00449-y

**Published:** 2023-07-12

**Authors:** Tai Li, Yi Jin, Jian Wu, Zhuqing Ren

**Affiliations:** 1 Key Laboratory of Agriculture Animal Genetics, Breeding and Reproduction of the Ministry of Education & Key Laboratory of Swine Genetics and Breeding of the Ministry of Agriculture and Rural Affairs, College of Animal Science, Huazhong Agricultural University, Wuhan, 430070 Hubei P. R. China; 2 Frontiers Science Center for Animal Breeding and Sustainable Production, Wuhan, 430070 China; 3 Hubei Hongshan Laboratory, Wuhan, China

**Keywords:** Lipid droplet, Embryo, Embryonic development, Lipophagy

## Abstract

Since the discovery, lipid droplets (LDs) have been recognized to be sites of cellular energy reserves, providing energy when necessary to sustain cellular life activities. Many studies have reported large numbers of LDs in eggs and early embryos from insects to mammals. The questions of how LDs are formed, what role they play, and what their significance is for embryonic development have been attracting the attention of researchers. Studies in recent years have revealed that in addition to providing energy for embryonic development, LDs in eggs and embryos also function to resist lipotoxicity, resist oxidative stress, inhibit bacterial infection, and provide lipid and membrane components for embryonic development. Removal of LDs from fertilized eggs or early embryos artificially leads to embryonic developmental arrest and defects. This paper reviews recent studies to explain the role and effect mechanisms of LDs in the embryonic development of several species and the genes involved in the regulation. The review contributes to understanding the embryonic development mechanism and provides new insight for the diagnosis and treatment of diseases related to embryonic developmental abnormalities.

## Introduction

Lipid droplets (LDs) are highly dynamic subcellular organelles that play an important role in lipid storage, metabolic homeostasis control, and the maintenance of dynamic stability in the intracellular environment [1]. They possess an outer monolayer consisting of phospholipids and proteins, encasing a neutral lipid core, such as triacylglycerols (TAGs), which is hydrophobic. Highly dynamic organelles exhibit significant morphological variations among different cells or at different metabolic levels. During cellular starvation, TAG lipase in the cytoplasm hydrolyzes LDs, a process called lipolysis. The resulting decomposed products then generate the energy required by the cell through the fatty acids (FAs) beta-oxidation of mitochondria [2].

Autophagy is an intracellular degradation process in eukaryotic cells that transports cytoplasmic components to lysosomes or vesicles for degradation or recycling. Furthermore, autophagy-mediated degradation of LDs, known as lipophagy, has been observed sequentially in mammals [3] and yeast [4], and it may also occur in plants [5]. Studies in yeast and animals have revealed that lipophagy serves various functions, including intracellular environmental homeostasis, energy production, and stress mitigation [2, 6–12].

Intracellular deposition of free fatty acids (FFAs) can have toxic effects, leading to impaired hepatocyte function and apoptosis [13–15]. When FFAs levels exceed the cellular metabolic capacity, resulting in an excess of FFAs, lipids are deposited intracellularly, releasing inflammatory factors that initiate apoptotic signals and sensitize cells to inflammation and injury [16]. This damage eventually leads to cell damage or apoptosis, a condition known as lipotoxicity [17]. Lipids that may be toxic to cells, such as cholesterol and FFAs, are sequestered within LDs [18]. Maternal nutritional overload can lead to lipotoxicity and oxidative stress. Excessive intake of nutrients such as fat and sugar will increase the risk of fetal fat deposition, fetal obesity, and a lipid metabolism disorder. These metabolites further trigger oxidative stress and a series of harmful reactions. Additionally, maternal nutritional overload may cause abnormal changes in the placenta, increasing fetal exposure to harmful substances and the potential for oxidative stress. Furthermore, nutritional overload can affect the normal function of the maternal immune system, inducing obesity-related inflammation and immune response, thereby exacerbating fetal oxidative stress and lipotoxicity [19]. During embryonic development, maternal nutritional overload can result in lipotoxicity, leading to oxidative stress and a decrease in the quality of embryonic development. However, the anti-stress ability of LDs stabilizes the cellular environment and significantly reduces the potential for cellular lipotoxicity, thereby ensuring normal cellular function.

LDs can be generated through various mechanisms and are dynamically connected to other organelles, facilitating the exchange of lipids and proteins [1, 20–25]. LDs serve as storage sites for diverse lipids that can act as signaling molecules or be converted into signaling molecules upon release. FFAs released from LDs can directly bind to cell surface receptors, triggering intracellular signal transduction pathways. These pathways involve peroxisome proliferator-activated receptors (PPARs), sterol regulatory element-binding proteins (SREBPs), and nuclear factor kappa-B transcription factors [26–28]. LDs actively store biologically active signaling molecules internally and influence signaling pathways by regulating their release and production of signaling molecules [28, 29]. Changes in intracellular gene expression during embryonic development suggest that genes play a regulatory role in embryogenesis and enable LDs to interact with other organelles.

In this review, we provide a comprehensive overview of current research in the field of LDs in embryos. We focus on elucidating the functional roles of LDs during different stages of embryonic development and explore the gene targets associated with LDs. Additionally, we discuss how understanding LD function and gene targets can contribute to advancements in vitro embryo culture and human in vitro fertilization (IVF) technology, providing new insights and ideas.

## The role of LDs in embryonic development

### The distributions of LDs in embryonic development

LDs are widely distributed in the reproductive system, and their abundance and distribution can be observed during embryonic development. Bik et al. employed near-infrared, mid-infrared, and Raman imaging techniques to investigate the distribution of LDs and other substances in fertilized Medaka Fish eggs [30]. Electron microscopy analysis of brown adipocytes in embryos revealed a potential association between LDs and glycogen [31]. Gupta et al. fixed zebrafish embryos at the cleavage stage using a 4% paraformaldehyde fixative and performed total internal reflection fluorescence (TIRF) imaging. Nile red-stained LDs were observed to accumulate at the cleavage groove using a 100×TIRF lens in an Olympus TIRF microscope. Similarly, Yang et al. employed the same approach to examine LDs in mouse Cumulus–oocyte complexes [32, 33].

Hallberg’s laser scanning microscopy observations showed significant changes in LipidTOX™ stained LD distribution during the cleavage of experimentally treated bovine oocytes [34]. To determine the nature of pro-Sudanese LDs in sheep blastocysts, this study used acetone to distinguish polar lipids from neutral lipids, which were soluble in acetone, and the complete absence of Sudan Black B staining was observed in light microscopy as LDs containing neutral lipids [35]. Tao et al. incubated the porcine embryo samples for 1 h and observed them under a confocal microscope and observed not only the distribution of LDs at the cleavage stage but likewise the number of LDs at the blastocyst stage [36].

LDs are present not only during the process of embryonic development but also in germ cells. Transmission electron microscopy observation of semi-thin sections of in vitro mature porcine oocytes subjected to electrical stimulation revealed a more uniform distribution of LDs in oocytes developing in vivo compared to those developed in vitro [37]. Microscopic examination of quail, duck, and turkey oocytes after induction of lipogenic differentiation also revealed the presence of LDs [38]. These observed results that LDs are pervasive throughout the development of life, not only in the oocytes but also in all stages of embryonic development, and may play a potential role in embryonic development.

### Multifunction of LDs in embryo

LDs may have multiple roles in the reproductive system, including energy storage and metabolism, lipid membrane conversion, signaling, mitigation of cellular stress, and temporary storage of proteins.

Within cells, FAs exhibit various destinies. In addition to their roles in membrane assimilation, lipid reserve storage, and as signaling molecules in lipid pathways, FAs can undergo oxidation, releasing energy and producing carbon dioxide and water as byproducts. Embryonic cells are adaptive and require appropriate amounts of FAs. As an indirect repository of intracellular FAs, LDs play an irreplaceable role in maintaining energy metabolic homeostasis. Cells use two main mechanisms to mobilize FAs during nutrient stress. One mechanism is autophagic digestion via membrane-bound organelles (i.e., the endoplasmic reticulum) or LDs [39–42]. This involves autophagosomes engulfing organelles/LDs and fusing with lysosomes, where hydrolases digest the organelles/LDs and release FFAs that rapidly enter the cytoplasm [3]. The second mechanism for the mobilization of FAs during starvation is through the lipolytic consumption of LDs. In this process, cytoplasmic neutral lipase directly hydrolyzes TAG on the LD surface. Mitochondria are the main site of β-oxidation, and during nutrient stress, FAs are catabolized by enzymes to maintain energy levels in embryonic cells.

The morphology and number of cytoplasmic LDs change during the maturation and fertilization of porcine oocytes [43]. There are significant differences in LDs in embryos before in vivo and in vitro implantation, which may be related to the energy requirements during porcine embryo development [37]. An LD was formed during the transformation of mouse embryonic stem cells (ESCs) to 2-cell stage embryo-like cells (2CLCs). Intriguingly, the glycolytic capacity and respiratory activity of 2CLCs are weaker than those of ESCs, and it is reasonable to assume that the ATP levels of 2CLCs are lower than those of ESCs. However, the difference in ATP levels between 2CLC and ESCs is not significant, indicating the potential to utilize a unique energy metabolic pathway [44].

Two degradation systems, the ubiquitin-proteasome pathway, and autophagy are required in early embryonic development to degrade maternal proteins and lipids into nutrients and raw materials for embryonic development. LDs are also required for normal embryonic development [45]. Using the autophagic degradation system expressing P62 as LD autophagic cargo to perform forced lipophagy is an intriguing approach. In this process, LDs aggregate and translocate to the cell periphery, leading to reduced viability of mouse embryos due to excessive energy depletion [46]. The zebrafish oocyte-to-embryo transition necessitates an additional ATP pulse to maintain the dynamic homeostasis of the embryo. Interestingly, instead of consuming the maternally provided yolk-FFAs pool and yolk-FACoA pool during this pulse preparation, LD-mediated lipolysis is utilized to provide the energy required to reach the pulse. This demonstrates the important role of LDs in supplying energy during early zebrafish embryonic development [47].

**Fig. 1:**
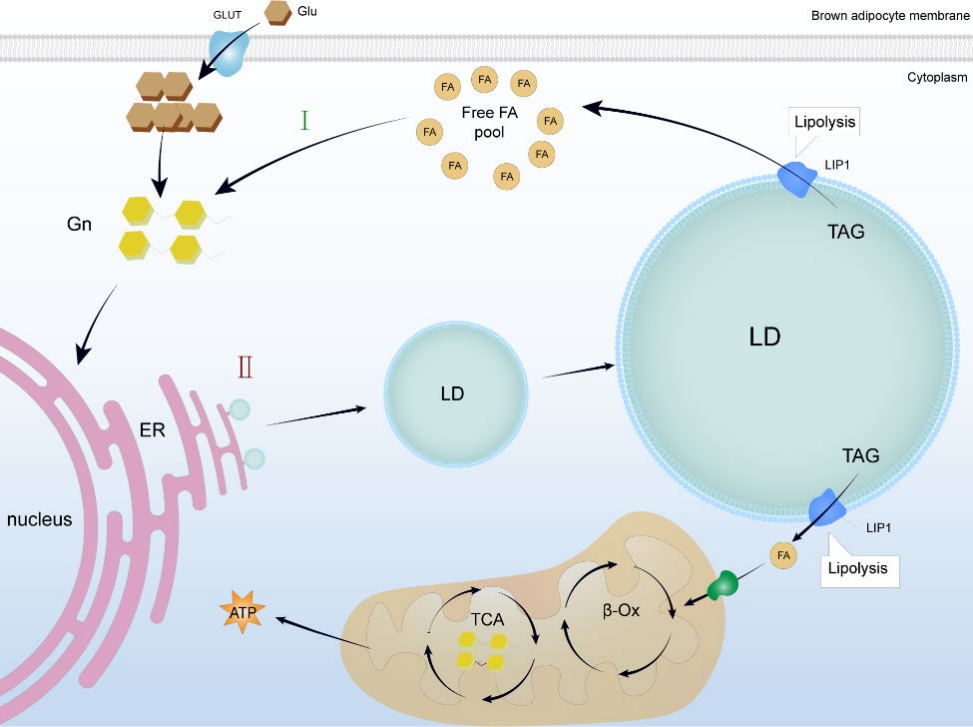
Intra-embryonic LD-glycogen conversion: FA, a TAG breakdown product within LDs, will be involved in gluconeogenesis to generate glycogen, which is biogenic to LDs during embryonic development. Green I: Gluconeogenesis. Red II: Biogenesis of LDs from glycogen. Abbreviations: LD, lipid droplet; FA, fatty acid; TAG, triacylglycerol; Glu, glucose; GLUT, glucose transporter; Gn, glycogen; ER, endoplasmic reticulum; TCA, tricarboxylic acid cycle; β-Ox, β-oxidation; ATP, adenosine triphosphate LIP1; lipase 1

**Fig. 2:**
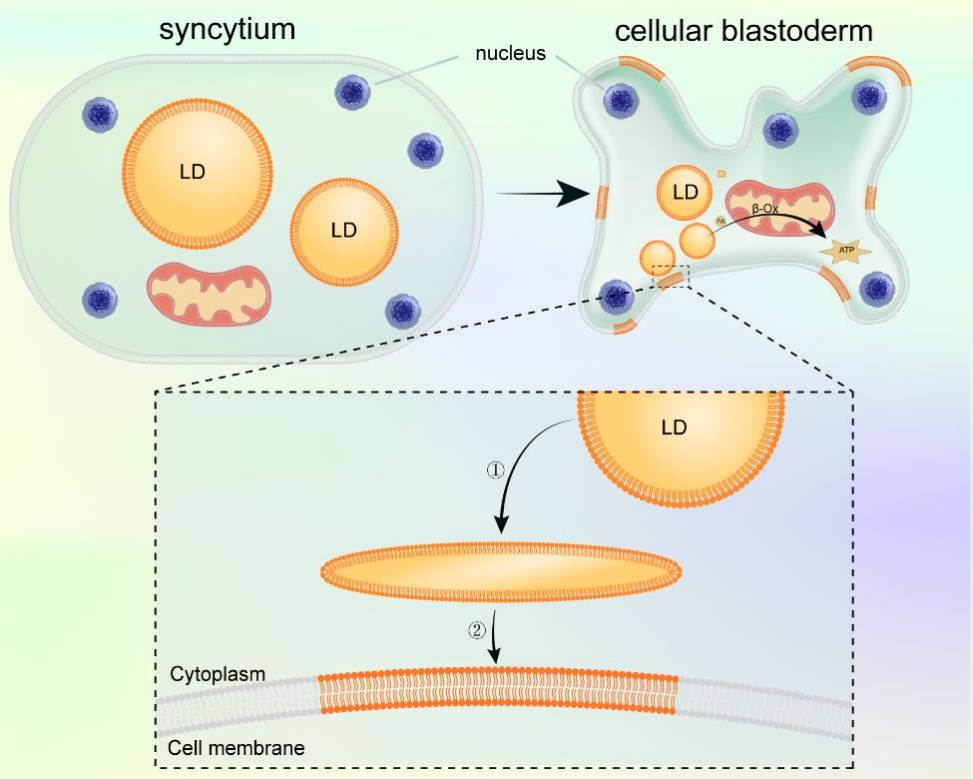
LD membranes are the source of membrane precursors for blastocyst cellularization. LD membranes gradually invaginate to become membrane precursors for the plasma membrane and other membrane structures during blastocyst cellularization division

Lipophagy does not necessarily directly convert to ATP for cellular energy supply. As the embryo develops, there is a decrease in lipids such as phospholipids and TAGs in the egg, while the opposite trend is observed in the glycogen near-infrared spectrum, suggesting that lipid consumption accompanies carbohydrate production [30]. Due to the biogenesis of LDs by glycogen during embryonic development, newborn rodents are filled with LDs after the first cold exposure [31]. These studies indicate that the interconversion of LDs and other nutrients during embryonic development ensures normal development (Fig. 1). LDs can serve as a source of membrane precursors for blastocyst cellularization (Fig. 2). Additionally, during differentiation, the plasma membrane invaginates and fuses into the nuclear membrane of the nucleus of the embryonic peripheral syncytium [48], with LDs playing a significant role in the composition of membrane components during cell differentiation.

LDs also play a role in alleviating cellular stress during embryonic development. Overfeeding during mare gestation did not affect the accumulation of LDs in the blastocyst during the first seven days, indicating that the physical condition of the mother does not immediately impact embryo development [49]. This is attributed to the stress-relieving effect of LDs. However, the ability of LDs to alleviate stress is also limited. Compared to control mice, mice fed high-fat diets showed significantly increased lipid accumulation in oocytes and enhanced endoplasmic reticulum stress, resulting in low fertilization and blastocyst rates and reduced embryonic developmental potential. Maternal hyperthermia during critical stages of embryonic development can lead to the accumulation of LDs in the trophectoderm, which may result in malformation or developmental delay in rat fetuses [50, 51].

Enhanced lipophagy of LDs in mouse cervical tissue may be controlled by progesterone. Lipolytic enzyme levels and LDs lipolysis in mid-pregnancy are closely related, suggesting that energy supply or hormonal facilitation is required to maintain cervical closure. Additionally, in a biotin-deficient environment, biotin, an essential vitamin for lipid and protein synthesis, cannot recruit TAGs into LDs [52]. The formation of LDs mitigates the extent of cell damage since excessive deposition of FFAs inside cells can cause lipotoxicity.

What would LDs do if embryos were in an environment filled with bacterial contamination? Bacterial contamination poses an extremely serious hazard to embryonic development. When simulating a bacterial infection environment, LDs in Drosophila play a key role in embryonic development. Not only do LD interconvert with glycogen in embryonic cells and provide energy for embryonic development through lipophagy, but their membrane components also act as membrane precursors for syncytium division. Additionally, LDs can temporarily store maternally supplied proteins and nutrients, preventing the degradation of excess histones before entering the nucleus and avoiding the onset of lipotoxicity. They can also load histones onto the LD, enhancing the defense against bacterial infestation [53]. Furthermore, a significant increase in the size of LDs was observed in a sample of female dogs with pus accumulation in the uterus [54].

**Fig. 3:**
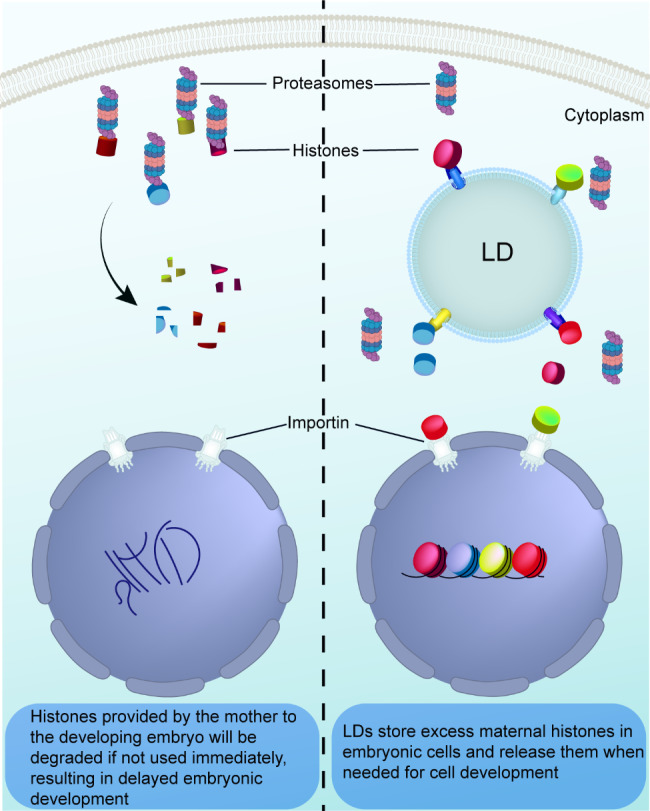
LDs are temporary reservoirs of free proteins. The proteins provided by the mother do not function immediately in the embryonic cells, and the LDs bind to them, thus avoiding the degradation of histones by intracellular enzymes. When these proteins are needed after embryonic cell division, the LDs release them

Intriguingly, maternal proteins are provided to the embryo, but some proteins are not immediately used during embryonic development, and LDs temporarily isolate proteins provided by the mother or proteins that are not immediately bound until the embryonic cells need them to supply effective proteins (Fig. 3) [55]. This suggests that LDs are not only good stress relievers and disease fighters for cells but also protein reservoirs that temporarily bind free proteins.

### The importance of LDs for embryonic development

In animal production and laboratory animal research, energy and nutrients are critical during embryonic development. Cumulus–oocyte complexes have a potential role in regulating TAG metabolism and β-oxidation processes produced by lipophagy, and elucidating this regulatory role could develop the potential for oocyte development in domestic animals [56]. Furthermore, extensive studies of embryonic in vitro culture techniques in animals have found that the presence of serum increases the abundance of neutral LDs, but the accumulation is heterogeneous. The reason for the uneven distribution is the result of the unique adaptation of LDs [35], as certain regions of the cell are more in need of LD participation, leading to a greater number of LDs in these regions.

The size and number of intra-embryonic LDs in B. indicus and B. taurus at the mulberry blastocyst stage affect the outcome of embryos after cryopreservation. The reason is that freezing is the denaturation of structural proteins on the LD surface, leading to the rupture of LDs, the outflow of their contents, and an increase in saturated fatty acids (SFAs) in the cytoplasm, causing lipotoxicity [57]. This finding provides a new strategy for the cryopreservation of embryos, exploring whether controlled lipolysis of LDs prior to preservation can increase embryo viability. Another approach is to load LD with specific proteins that are resistant to cold stress, aiming to prevent LD rupture due to low temperature and improve the pregnancy rate. This idea also opens up new possibilities for the preservation of blastocyst stage embryos.

For human reproduction, maternal rats with obesity and alcohol consumption have a higher incidence of congenital heart disease in their offspring than normal individuals due to the abnormal formation of LDs as a result of abnormal placental lipids [58]. Lipophagy in patients with advanced ovarian cancer is non-regulatory, and cancer cells enhance lipophagy to provide energy for their proliferation, and inhibition of lipophagy can effectively inhibit cancer progression [59]. These studies show that the health status of the mother has an impact on the development of the offspring, which sheds light on the implications for human diet and disease during pregnancy preparation, and even the role of LD as a temporary repository of proteins to specifically bind anti-cancer proteins and thus help in cancer treatment, which is not only a potential way to save mothers with cancer from reproducing offspring properly but also a new way of thinking about the fight against cancer.

In vitro embryo culture technology plays an important role in livestock production, and the quality of embryo development affects productivity. Treatment of in vitro embryos with appropriate concentrations of melatonin has been shown to increase the number of LDs while decreasing their size, improving oocyte quality and providing necessary energy for embryonic development. Feeding fish oil to broiler females during gestation can reduce fat deposition in the offspring and reduce the potential risk of obesity in the offspring [60]. The effect of adding a synthetic serum to cultured embryos was similar to that of adding fetal calf serum [61], and the serum did not cause lipid accumulation or organelle damage to bovine embryonic cells [62], reducing the concentration of fetal bovine serum to 5% for culturing in vitro embryonic cells is an appropriate concentration [63]. Supplementation with all-trans retinoic acid [64], L-carnitine [65], melatonin [66], and dehydroepiandrosterone [67] in serum can improve in vitro embryo culturing, which provides a method for the livestock industry including other fields that require embryo culturing in vitro, and hopefully, more serum additives will enhance embryo In vitro culture viability. However, human in vitro embryo culture is still in the IVF period, and there are many difficulties in the late in vitro culture technology. Experimental findings obtained from animal studies may help address the challenges of in vitro culture of human embryos, representing a significant potential breakthrough.

## LDs-related genes and regulatory functions in embryonic development

### Signal pathways in embryonic development

Regulation of the AMPK pathway is essential for energy metabolism during embryonic development. AMPK is a heterotrimeric complex consisting of three subunits, which β and γ as catalytic subunits and α is a regulatory subunit [68]. AMPK activity can be activated by kinases and pharmacological activators, leading to the activation of AMPK subunits [69]. The intracellular AMP/ATP ratio influences the activity of AMPK. When AMP/ATP levels are elevated, AMPK is activated, inhibiting lipid synthesis and promoting FA oxidation [70, 71]. AMPK acts as a stress response in conditions of energy deficiency and oxidative stress. It phosphorylates target proteins to regulate lipid metabolism [72]. Acetyl coenzyme A carboxylase (ACC) is a key enzyme involved in the synthesis of long-chain FAs, specifically encoded by acetyl coenzyme A carboxylase 1 [73]. During oocyte development, the ACC gene promotes the potential for oocytes to differentiate into adipocytes [66]. Activated AMPK phosphorylates and inactivates ACC, reducing FA synthesis through the AMPK/ACC pathway. This promotes the expression of carnitine palmitoyltransferase 1(CPT1) and increases FA oxidation. The ACC gene plays a significant role in LD formation, and its reduced activity affects the TAG within LDs, thus impacting energy storage and metabolism [74, 75]. Expression of ACC and CPT1 genes provides the energetic foundation for subsequent embryonic developmental processes. During development, if there is nutrient overload, embryonic cells are regulated through the PI3K/AKT pathway. The production of 3’-phosphorylated phosphatidylinositol activates the PI3K/AKT pathway, which may regulate the expression of certain lipid genes during embryonic development [76].

Fatty acid synthase (FASN) is a key enzyme involved in the de novo synthesis of FAs. It catalyzes the production of palmitate and 16-carbon long FAs from acetyl coenzyme A and malonate coenzyme A [77]. SREBP1C activates FASN by binding to its promoter region, with contains sterol regulatory elements [78]. Studies have shown that PI3K/AKT signaling pathway influences the expression of SREBP1C/FASN, regulating the conversion of glycolipids in cells in response to sugar concentration [79]. This process may involve the biogenic effects of glycogen on LDs, leading to increased FASN levels and promoting LD synthesis. Consequently, energy is converted into lipids for storage in LDs, helping to regulate nutrient levels during embryonic development.

The Wnt pathway plays a crucial role in regulating stem cell pluripotency and determining cell fate during embryonic development. It also controls the formation of the embryonic axis, axons, organs, and other important processes during embryonic development. The components of the Wnt pathway include Wnt ligands, G protein-coupled transmembrane frizzled receptors, and low-density lipoprotein-related receptor co-receptors [80]. Monounsaturated fatty acids (MUFAs) activate the Wnt/β-catenin pathway, transmitting signals to the nucleus [81], A similar process may occur during embryonic development. Three distinct isoforms of SREBPs, namely SREBP1a, SREBP1c, and SREBP2, are expressed in various human tissues. These isoforms are encoded by separate genes [82]. The SREBP1c isoform primarily governs FA synthesis, while SREBP2 regulates genes involved in cholesterol biosynthesis and embryonic development. Interestingly, the SREBP1a isoform is involved in both lipogenic pathways [83–85]. Depletion of SREBP1 leads to decreased levels of unsaturated lipids and triggers apoptotic cell death when cells have limited access to exogenous lipids. Activation of the Wnt/β-catenin pathway induces SREBP-1c to activate genes necessary for FA and triacylglycerol synthesis, such as stearoyl-CoA desaturase 1 (SCD1).

SCD1 is an integral protein located in the endoplasmic reticulum membrane. It catalyzes the synthesis of polyunsaturated FAs, such as oleic acid (C18:1) and palmitoleic acid (C16:1), from FAs like palmitic acid (C16:0) and stearic acid (C18:0). This enzymatic activity introduces cis double bonds between carbons 9 and 10 of the stearic acid and palmitic acid. Oleic acid, an unsaturated FA, is a significant byproduct of SCD1 activity. Intriguingly, the extent of exogenous oleic acid supplementation positively affects LD formation in embryos. In contrast, the presence of saturated FAs has detrimental effects on both embryo development and LD formation [86]. This highlights the critical role of SCD1 in early embryonic development and emphasizes its importance in this intricate process.

### Genes regulating LDs formation in the embryo

LDs in the embryo require proper gene expression regulation to function. Perilipin, a core LD-associated protein, is well-known for its important role in lipid metabolism. Recent studies have also identified other crucial genes involved in lipid metabolism. Among these genes, PPARs are members of the intranuclear receptor transcription factor superfamily that regulate the expression of target genes. PPARα, in particular, is a significant nuclear receptor that controls the expression of the CPT1 gene, which is involved in FA catabolism [87]. The activity of PPARγ affects the formation of LDs in adipocytes [88]. Long-chain lipid CoA synthase is a key player in body lipid metabolism and is associated with various diseases. Specifically, long-chain acyl-CoA synthetases 1 (ACSL1) is a target gene of PPARα and co-regulates lipid metabolism in the body. SCD1 is a rate-limiting enzyme that converts SFA to MUFA and plays a role in LD formation through phospholipid formation.

DGAT1, CD36, or NR1H3 have been identified as markers associated with lipids in porcine and bovine blastocysts. These genes are involved in LD synthesis [89]. Intriguingly, blocking the very long chain fatty acid enzyme 5(ELOVL5) gene reduced the expression of related lipids and promoted intracytoplasmic LD deposition in blastocysts. However, this blockade did not affect embryo development or blastocyst cell number [90], possibly due to compensatory effects of other ELOVL family genes on lipid metabolism [91]. In nematode embryos, the SEIPIN-1 pair controls LD size and lipid homeostasis. Mutations in SEIP-1 lead to dysregulation of the lipid-permeable membrane in the innermost layer of the embryonic eggshell, resulting in embryonic death. However, supplementation with polyunsaturated FAs can resolve this issue [92]. Other genes associated with LD function in the embryo include GPI [93], LSD [94], Myosin family [32], and other genes regulated.

### LD-related genes contribute to embryonic development

Diacylglycerol Acyltransferase (DGAT) is the acyl-coenzyme A required to catalyze the final step of TAG synthesis. The genes encoding two DGAT enzymes, DGAT1 and DGAT2, were discovered long ago [95]. Changes in DGAT2 expression during oocyte formation suggest increased lipid synthesis in oocytes [63]. TAG stored in LDs serves as backup energy for the first embryonic cleavage [96]. The expression of DGAT2 and ACC genes provides the energy basis for subsequent embryonic development.

Myosin-1(Myo1) is a motor protein involved in early embryonic development. It binds to other motor proteins and is recruited to specific binding sites involved in membrane invagination and rupture [97]. Intriguingly, zebrafish oval spheres contain dynamic LDs, and embryos with suppressed Myo1 show an accumulation of LD in the sulcus line [32]. Myo1 is involved in maintaining the oval sulcus and driving the movement of LDs. ACSLs modify FFAs by catalyzing the formation of Acyl-CoA and activating them. TriacsinC treatment of non-defatted embryos leads to substantial LD degradation. Other drugs that promote lipase activity do not reduce LD intensity, but ACSL activity reduction leads to embryonic developmental defects [98]. These results suggest that ACSL activity is crucial for the synthesis and maintenance of LDs and is a key factor in LD biogenesis.

**Fig. 4:**
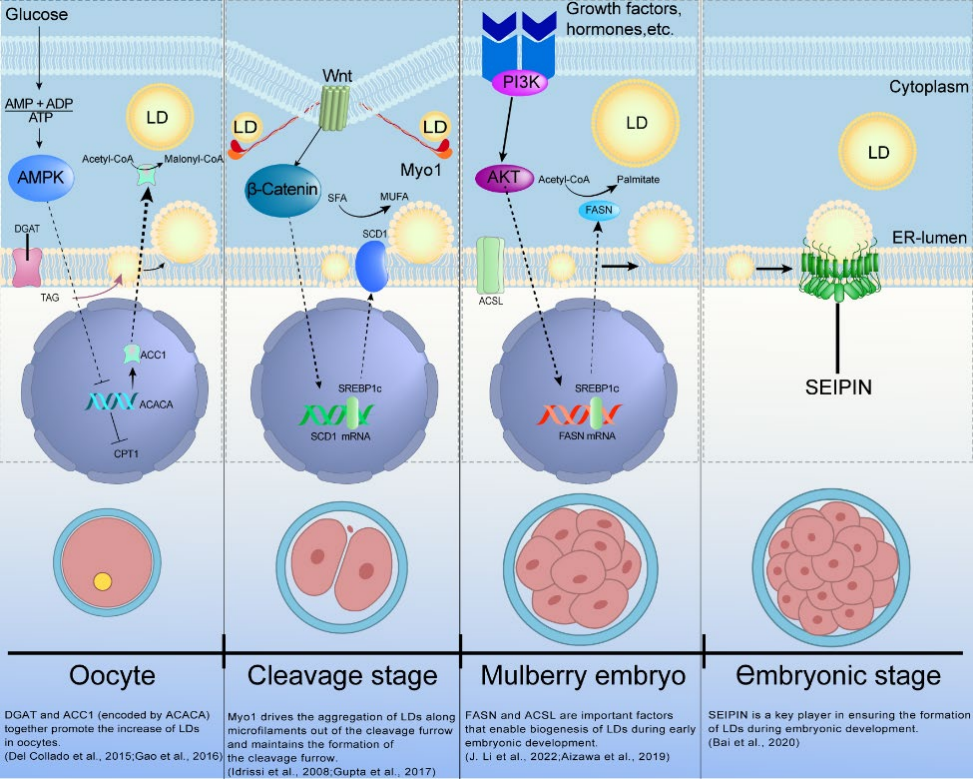
Signal pathways associated with LDs at various stages of embryonic development. In the oocyte, the ACACA gene encodes acetyl coenzyme A carboxylase 1 by the AMPK pathway, which then enters the cytoplasm and converts Acetyl-CoA to Malonyl-CoA. DGAT synthesizes TAGs to encapsulate LDs. During the cleavage stage, Myosin1 drives LDs to the cleavage groove and maintains the formation of the cleavage groove by Wnt/β-catenin. SCD1 catalyzes the conversion of SFA to MUFA. During the mulberry embryo period, ACSL converts long-chain FAs to acyl-CoA by PI3K/AKT pathway. SEIPIN is an important gene for LD biogenesis throughout embryonic development. These genes may function at other stages of the embryo, and the timeline is based on available studies

SEIPIN is an evolutionarily conserved protein encoded by the Berardinelli-Seip congenital lipodystrophy 2 gene. It is localized to the endoplasmic reticulum [99, 100] and plays a key role in biogenesis. During Caenorhabditis elegans embryo development, SEIPIN1 is involved in forming the permeability barrier, which protects the embryo from toxic molecules and damage. SEIPIN1-deficient Caenorhabditis elegans mutants disrupt the dynamic balance of FFAs in embryos, leading to embryonic death [92]. This suggests that SEIPIN1 plays a critical role in FFA storage during embryonic development. Genes associated with LDs regulate the internal environment of embryonic development during the various processes of embryonic development, ensuring proper embryonic development and positioning of embryo culture at the genetic level (Fig. 4).

### LD-related genes can be potential genetic targets during embryonic development

Whether for improving embryonic development in vivo or applying it to in vitro embryo culture techniques, regulating the expression of the aforementioned genes may yield the desired results. PPARs are important nuclear receptors that regulate FA metabolism in the body. By controlling the upstream control genes of PPARs, lipid metabolism can be improved in embryos with severe fat deposition, creating an ideal cellular environment for embryonic development. PPARs can play a key role in regulating the overall development of embryonic tissues. CPT1 and ASCL1 are target genes of PPARα, and the expression status of these pathways directly impacts lipid metabolism in the embryo. DGAT and ACC are key enzymes involved in the synthesis of long-chain FAs and are closely related to LD production. Interestingly, Myo1 is a key gene in embryonic cell division [101]. Exploring the effect of Myosin gene family expression on the rate of embryonic cellularization would be an interesting direction to investigate, as it may facilitate the process of embryonic development.

In conclusion, these genes associated with LDs will be favorable targets for studying the functional expression of LDs in the embryo and exploring their regulation will be of great significance for embryo development and culture techniques.

## Conclusion and prospect

The current understanding of the involvement of LDs in embryonic development remains in its nascent stages despite ongoing attempts to explicate their role. The extant literature on LDs and their associated genes has primarily concentrated on their functions in the liver, adipose tissue, and macrophages. This review seeks to address this gap in knowledge by elucidating the function of LDs and related genes in the context of embryonic development.

Due to the biological intricacies of embryonic development, the lipid composition is a crucial aspect that needs to be considered. Further investigations into the role of LDs in the embryo can provide additional insights into their importance. A deeper understanding of the interconversion of LDs with other nutrients, such as proteins, is necessary to comprehend the influence of LDs on embryonic development. Additionally, it is pertinent to investigate the process of embryonic cellularization, which involves the formation of membrane precursors from LDs, and the storage and release of proteins on these precursors. The mechanisms of protein storage and release, as well as the regulation of DGAT during embryonic development, also warrant further exploration. Furthermore, it is essential to investigate whether modulating Myo1 protein can accelerate LDs’ movement along microfilaments to the cleavage groove and promote the cleavage process. Additionally, studying the impact of serum concentration on LD levels in cells through in vitro embryo culture techniques is also necessary. By addressing these questions, we can overcome the challenges posed by in vitro embryo culture technology in animal production and enhance production efficiency. Additionally, addressing these queries may result in breakthroughs in human IVF technology and may extend the duration of in vitro culture. Furthermore, resolving these queries can provide therapeutic targets for the treatment of embryos with maternal obesity and other inflammatory diseases.

## Data Availability

Not applicable.

## Abbreviations

LDs: Lipid droplets
TAG: Triacylglycerol
FAs: Fatty acids
FFAs: Free fatty acids
PPARs: Peroxisome proliferator-activated receptors
SREBPs: Sterol regulatory element-binding proteins
IVF: In vitro fertilization
TIRF: Total internal reflection fluorescence
ESCs: Embryonic stem cells
2CLCs: 2-cell stage embryo-like cells
ACC: Acetyl coenzyme A carboxylase
CTP1: Carnitine palmitoyltransferase 1
FASN: Fatty acid synthase
MUFAs: Monounsaturated fatty acids
SCD1: Stearoyl-CoA desaturase 1
ACSLs: Long-chain acyl-CoA synthetases
ACSL1: Long-chain acyl-CoA synthetase 1
DGAT: Diacylglycerol Acyltransferase
Myo1: Myosin-1
